# EffEx-HN trial: study protocol for a randomized controlled trial on the EFFectiveness and feasibility of a comprehensive supervised EXercise program during radiotherapy in Head and Neck cancer patients on health-related quality of life

**DOI:** 10.1186/s13063-023-07170-x

**Published:** 2023-04-15

**Authors:** Kaat Van Aperen, An De Groef, Nele Devoogdt, Tessa De Vrieze, Thierry Troosters, Heleen Bollen, Sandra Nuyts

**Affiliations:** 1 Department of Oncology, Laboratory of Experimental Radiotherapy, University of Leuven, Leuven, Belgium; 2 Department of Rehabilitation Sciences and Physiotherapy, University of Leuven, Leuven, Belgium; 3 Department of Rehabilitation Sciences and Physiotherapy, MOVANT Research Group, University of Antwerp, Antwerp, Belgium; 4 Department of Physical Medicine and Rehabilitation, University Hospitals Leuven, Leuven, Belgium; 5 Department of Vascular Surgery, University Hospitals Leuven, Leuven, Belgium; 6 Respiratory Rehabilitation and Respiratory Division, University Hospitals Leuven, Leuven, Belgium; 7 Department of Radiation Oncology, Leuven Cancer Institute, University Hospitals Leuven, Leuven, Belgium

**Keywords:** Quality of life, Exercise, Cancer rehabilitation, Head and neck cancer, Radiotherapy, Effectiveness, Feasibility, Tele-coaching

## Abstract

**Background:**

With over 500,000 annually reported cases worldwide, head and neck cancer (HNC) is the seventh most common type of cancer worldwide. Treatment of HNC with chemoradiotherapy frequently results in serious impairments in physical and psychosocial functioning. Besides, HNC patients typically start their cancer treatment already with poor physical and psychosocial health. It has been shown that a sufficient level of physical activity (PA) before, during, and after cancer treatment is associated with fewer negative treatment-related side effects and a better quality of life (QOL). In order to prevent worsening of functioning and limit the physical impact of the HNC treatment, a comprehensive supervised exercise program (CSEP) may be beneficial during early cancer treatment. However, up to now, the feasibility and effectiveness of such a program are not yet investigated thoroughly in HNC. Therefore, the primary objective of this study is to examine the effectiveness of a CSEP during HNC treatment, in addition to usual supportive care, compared to usual supportive care alone, on health-related QOL up to 1 year post-diagnosis. Secondary objectives entail gathering information on (1) the effectiveness of a CSEP on secondary outcomes such as physical and mental function, activities of daily life, and participation in society and (2) the feasibility, possible barriers, and facilitators for participation in a CSEP during HNC treatment.

**Methods:**

To investigate the effectiveness of the CSEP, a parallel, open-label randomized controlled trial will be performed. To study the feasibility of the CSEP, a mixed-method study will be performed in a subgroup of participants. HNC patients are eligible if they receive radiotherapy at the Radiation-Oncology department of the University Hospital of Leuven. A 4-size permuted block randomization will be used. The control group receives the current standard of supportive care. The intervention group receives a CSEP, additional to the same usual supportive care. The CSEP consists of a 12-week intensive phase with 3 exercise sessions of 1 h per week, where supervision is gradually reduced after 6 weeks. During the maintenance phase (from week 13), patients exercise at home with monthly tele-consultations with a physiotherapist. The CSEP contains supervised aerobic and resistance training. In both groups, outcomes of interest are evaluated through self-reported questionnaires and clinical assessments, at baseline, 6 weeks, 12 weeks, 6 months, and 12 months post-diagnosis. The primary endpoint is health-related QOL, measured with the EORTC QLQ-C30 at 6 months post-diagnosis.

**Discussion:**

The study will be conducted in accordance with the Declaration of Helsinki. This protocol has been approved by the ethical committee of the University Hospitals Leuven (s65549). Recruitment started in January 2022. Results will be disseminated via peer-reviewed scientific journals and presentations at congresses.

**Trial registration:**

Trial Registration: ClinicalTrials.gov

Identifier: NCT05256238

Date of registration: February 25, 2022

## Administrative information

**Table:** 

Title	EffEx-HN Trial: Study protocol for a randomized controlled trial on the effectiveness and feasibility of a comprehensive supervised exercise program (CSEP) during radiotherapy in head and neck cancer patients on health-related quality of life
Trial registration	ClinicalTrials.gov Identifier: NCT05256238 February 25 2022
Protocol version	Version 2.2 January 20, 2022
Funding	Kom Op Tegen Kanker
Author details	sandra.nuyts@uzleuven.be + 32 16 347 600 an.degroef@kuleuven.be + 32 16 342 171 kaat.vanaperen@kuleuven.be + 32 16 345 196
Name and contact information for the trial sponsor	University Hospitals Leuven Herestraat 49, 3000 Leuven, België + 32 16 33 22 11
Role of sponsor	The study is conducted at the University Hospitals Leuven

## Introduction

### Background and rationale

With over 500,000 annually reported new cases worldwide, head and neck cancer (HNC) is the seventh most common type of cancer worldwide [1, 2]. HNC contains a diverse group of malignancies originating from the mucosa in the oral cavity, nasopharynx, oropharynx, hypopharynx and larynx. Unfortunately, the majority of patients with HNC have locally advanced disease at diagnosis and are, therefore, treated with chemoradiotherapy (CRT) or with radical surgery followed by adjuvant (C)RT. Treatment of HNCs with (C)RT frequently results in serious and persistent impairments in physical and psychosocial functioning [3, 4].

The WHO International Classification of Functioning, Disability and Health (ICF) model offers a framework for understanding disability and health, e.g., during and after HNC [5]. At function level of the ICF, swallowing and speech impairments are specific morbidities experienced in over half of HNC patients at various intensities before, during, and after cancer treatment [3]. Additionally, a large part of HNC patients experience lymphedema, pain, stiffness, and/or weakness of the jaw, neck, and shoulder [3, 4]. These local impairments are mainly caused by radiotherapy-induced fibrosis and neck dissection surgery. All this contributes to activity limitations in neck and shoulder function in about two thirds of HNC patients [3, 4]. More general consequence including fatigue, decreased physical fitness, muscle wasting, and related weight loss are other activity limitations [4–6]. These impairments restrict physical functioning and participation in activities of daily living of many patients, consequently impeding their quality of life [6]. Besides the physical and psychosocial consequences of the cancer treatment, HNC patients typically start their cancer treatment already with poor physical and psychosocial health, compared to healthy controls, and hence, QOL is already impaired prior to starting any intervention [6, 7].

The awareness on the importance of exercise therapy for management of the side effects at the different levels of the ICF during and after cancer treatment in general is increasing rapidly [6–9]. It has been shown that a sufficient level of physical activity before, during, and after cancer treatment is associated with a better prognosis, a lower risk of recurrence, a lower risk of mortality [7–10], fewer negative treatment-related side effects [9], and a better quality of life [8–11]. At this moment, the guidelines of the American College of Sports Medicine recommend 2- or 3-weekly 20–30 min sessions of moderate aerobic exercise plus 2-weekly resistance training at moderate to vigorous intensity (2 sets of 8–15 repetitions at > 60% of the 1 repetition maximum for major muscle groups) to have effect of exercise on health-related quality of life in cancer populations. These guidelines are based on research in mostly breast, prostate, and colon cancer populations as these are the most prevalent populations [8–10].

Up to now, it has not yet been investigated properly whether these general guidelines for exercise programs are translatable to the HNC population. Given the specific physical and psychosocial needs in the HNC population described above, this should be investigated properly [6–9]. In addition, since HNC patients often start their treatment already with poor physical functioning [6], exercise therapy should be initiated at time of diagnosis and continue during and after treatment. In the currently available studies, the outcomes and the content of the exercise programs are very heterogeneous making it difficult to draw conclusions at this moment [8–11]. First, in a pilot study of Zhao S.G. et al. from 2016, HNC patients undergoing concurrent CRT and participating in a program designed to maintain physical activity during cancer treatment maintained or improved function and QOL. They also concluded that the exercise intervention was feasible, thereby providing the basis for larger future interventions with longer follow-up [12]. Second, a study of Samuel et al. (2019) showed that an 11-week structured exercise program for HNC patients receiving CRT helps in improving their functional capacity and quality of life up to 11 weeks after diagnosis [13]. The exercise program consisted of 5 trainings per week, with 7 weeks in the hospital and 4 weeks of home-based trainings. Limitations of this study are that there was no maintenance program and no follow-up period after the 11 weeks.

In addition, next to the effectiveness of an exercise program on different health-related outcomes, barriers and facilitators for participating in a CSEP starting from diagnosis should be properly investigated. In the trial of Samuel et al., patients attend 75% of the prescribed sessions [14]. Unfortunately, specific barriers and facilitators or reasons for non-adhering were not studied. Possibly, the intense schedule of 5 trainings per week may have decreased adherence in that study. Also, it may be interesting to explore whether the combination of in hospital with home-based trainings is more beneficial. Travel time to the hospital is indeed a barrier to exercise, which confirms the need of combining hospital and home-based exercise programs [14].

Given this, a comprehensive supervised exercise program (CSEP) starting early during CRT may be beneficial to prevent worsening of a person’s functioning and limit the physical impact of the treatments for HNC [6–9]. Such a program should consist of a combination of aerobic training, progressive resistance training for all major muscle groups and stretching exercises, in particular in the upper limb region. The program should combine in hospital training with home-based trainings and have a sufficiently long duration.

### Objectives

There is a need to improve the integration of exercise programs into HNC care. The aim is to help HNC patients to prevent decline in and restore physical, mental and social functioning. Currently, no strong evidence is available for a program adapted to the specific needs of HNC patients, in particular during cancer treatment [3, 4, 6]. The primary objective is to examine the effectiveness of a CSEP, in addition to usual supportive care, on health-related quality of life during the treatment of HNC, compared to usual supportive care alone, up to 1 year after diagnosis. This will be performed through an open-label randomized controlled trial.

Secondary objectives entail gathering information on the effectiveness of the CSEP, in addition to usual supportive care on secondary outcome parameters including physical and mental functioning, activity level, and participation level of the ICF. In addition, the feasibility of such CSEP as well as possible barriers and facilitators for participating in a CSEP during HNC treatment will be studied.

### Trial design

A parallel, two-arm, open label trial will be conducted. This single center prospective randomized clinical study will be performed to investigate the effectiveness and the feasibility of a comprehensive supervised exercise program (CSEP) in addition to usual supportive care compared to usual supportive care alone in patients undergoing CRT for HNC, the EffEx-HN trial. This is a superiority trial design because we aim to investigate whether a CSEP is more effective in improving health-related quality of life than the current standard of supportive care. To study the feasibility of the CSEP, a qualitative study will be performed in a subgroup of participants of the large prospective randomized clinical study described above. Quantitative data on feasibility will be collected in all participants.

## Methods: participants, interventions, and outcomes

The SPIRIT guidelines were used [15].

The recruitment of the clinical trial started in January 2022 at the department of Radiation-Oncology of the University Hospitals in Leuven (Belgium). The supervised exercise sessions of the intervention group take place in the exercise room of the department of Physical Medicine and Rehabilitation of the University Hospitals in Leuven (Belgium). A flowchart of the EffEx-HN Trial is provided in Table 1.

**Table 1:** Trial flowchart: enrolment, intervention, and assessments of the EffEx-HN trial

Time points	0–1 weeks after diagnosis	*T*_0_ baseline (within 1 week after start of CRT)	*T*_1_ 6 weeks	*T*_2_ 12 weeks	*T*_3_ 6 months	*T*_4_ 12 months
**Enrolment**						
Eligibility screening	X					
Informed consent (intake session)	X					
Randomization	X					
Allocation	X					
**Intervention**						
Start CSEP (IG^a^)		X				
Start maintenance program with monthly tele-consult (IG^a^)				X		
**Assessments**						
Baseline assessment		X				
Follow-up assessments			X	X	X	X
Primary endpoint^b^					X	
Secondary endpoints^c^			X	X	X	X

^a^IG = intervention group ^b^Primary endpoint = 6 months post-diagnosis: primary endpoint for health-related quality of life ^c^Secondary endpoints = 6 weeks, 12 weeks, 6 months, and 12 months post-diagnosis for all outcome measures and 12 weeks for feasibility outcomes

### Patient and public involvement in trial design

The protocol was submitted and approved by the patient support group of HNC patients of the University Hospitals Leuven.

### Eligibility criteria

HNC patients are eligible to participate if they are scheduled for radiotherapy at the Radiation-Oncology department of the University Hospitals Leuven. Inclusion criteria are as follows: (1) ≥ 18 years, (2) diagnosed with primary malignant tumor head and neck region, (3) ECOG performance score 0–1, (4) able to complete baseline assessments prior to start of (chemo)radiotherapy, (5) physically and mentally capable of taking part in an exercise program and motivated to engage in a supervised exercise program. Patients are excluded if one of the following exclusion criteria are present: (1) < 18 years, (2) ECOG performance score ≥ 2, (3) treated with palliative intent, (4) evidence of distant metastases, (5) no basic level of reading and writing in Dutch. Participation of a patient in the study can be discontinued based on the participant’s request or if the disease is worsening, to the extent that exclusion criteria are present (treatment with palliative intent, evidence of distant metastases).

### Participant screening, recruitment, and consent

Participants are identified from scheduled consultation lists and screened for eligibility criteria. Potentially eligible participants are approached and recruited during the consultation at the department of Radiation Oncology of the University Hospitals Leuven, where the oncologist (principal investigator (PI)), SN, and patient discuss the suggested treatment option. The PI will inform every new patient, diagnosed with HNC eligible for this study, about the study. All eligible patients also receive a one-on-one explanation of the study by a member of the research team and an information letter during this consult. After obtaining informed consent, patients will randomly be assigned to receive either the usual supportive care + CSEP (intervention group) or usual supportive care only (control group).

### Allocation and randomization

A 4-size permuted block randomization is used. The randomization is computer-generated, in particular the allocation to the intervention or the control group is concealed and performed by the randomization module of the Research Electronic Data Capture system (REDCap) before the start of the exercise program, ensuring blinding of the research team [16, 17]. The sequence of randomization is decided by the patient’s record identifier in REDCap, which is received after signing informed consent. An open label study is performed, which means that the therapist/assessor and patients are not masked for the allocation to the intervention or control group. The therapist giving the exercise program and the assessor are the same individual during the entire study period.

### Interventions

#### Usual supportive care

Both groups will receive the current standard of supportive care including whenever required guidance by a dietician on oral food intake, smoking cessation counseling, and speech and language therapist guidance concerning swallowing exercises, follow-up by the social workers, psychologists, and nurse care team. Additionally, they will be informed at the start of treatment about the importance of physical activities, and a brochure with general advice for exercises will be provided.

#### Dedicated comprehensive supervised exercise program (CSEP)

The intervention group will receive the CSEP, additional to usual supportive care. The CSEP consists of a one-hour individualized exercise program three times a week, starting within 1 week after the start of the CRT, taking into account motivation, personal goals, and pre-diagnostic activity level. The program is tailored to the acute effects of CRT as well (e.g., intensity and number of sessions). The training sessions will be organized in small groups of 3–4 patients, consisting of a combination of 30 min aerobic training at moderate intensity (walking, cycling) and 30 min resistance strength training and stretching (exercises for all major muscle groups and in particular the upper limb, head and neck region). A total of 18 supervised sessions are held in the hospital twice a week in week 1 to 6 and once a week in week 7 to 12, while the remaining one or two weekly sessions are performed independently at home. During the maintenance program (from week 13), patients will exercise at home with a monthly tele-consultation. Patients will have online access to videos of exercises to be performed at home. A selection of exercises will be made for each individual patient based on preferences and tailored to their exercise tolerance. During the tele-consultation, the physiotherapist discusses the progression of the training program at home and if any adjustments are needed.

The CSEP is supervised by a physiotherapist, KVA. The therapist will be dedicated full time to this program to conduct the patient assessments, to supervise the exercise sessions at the hospital and the tele-consultations during the maintenance program, and to coordinate the project. In addition to the exercise program, the participants in the intervention group attend in the first month an educational session about exercise during and after cancer treatment.

### Outcomes

#### Effectiveness study

At 5 time-points, evaluation moments are organized, during which self-reported questionnaires and clinical assessments are performed. The evaluation moments take place at baseline (T0), at 6 weeks (T1), at 12 weeks (T2), at 6 months (T3), and at 12 months (T4). The assessments will be performed at the Department Physical Medicine at the University Hospitals Leuven. Most of the questionnaires are conducted at home, through a digital link to the Research Electronic Data Capture (REDCap). The evaluation moments take place in the hospital. In total, administering the questionnaires through REDCap takes 60 min. The clinical assessment takes 45 to 60 min. The primary outcome parameter is health-related QOL measured with the European Organisation for Research and Treatment of Cancer quality of life questionnaire (EORTC QLQ-C30) at 6 months post-diagnosis. A key-secondary outcome is the physical functioning subscale of the EORTC QLQ-C30. The secondary outcome measures are also shown in Tables 2 and  3. In addition, personal factors, including patient-related factors (age, social status, work status), and cancer (treatment)-related factors will be collected from the participant’s medical file.

**Table 2:** Study outcome measures by assessment time-point: self-reported outcomes

Domain	Questionnaire	*T*_0_	*T*_1_	*T*_2_	*T*_3_	*T*_4_
**Quality of life**						
Health-related quality of life (primary outcome)	EORTC QLQ-C30 [18] The EORTC QLQ-C30 is developed to assess the quality of life of cancer patients. The raw scores will be transformed to scores from 0 to 100 with 0 a worse health-related quality of life.	X	X	X	X	X
Disease-specific health-related quality of life	EORTC QLQ-HN43 [19] The EORTC QLQ-HN43 is a revised version specifically designed for HNC patients and used to measure the specific HNC symptoms. The scores range from 0 to 100. In functional scales, high scores represent a high level of functioning, while in symptoms, they represent a high level of symptomatology.	X	X	X	X	X
**Physical function**						
Pain	Brief Pain Inventory short form (BPI) [20] The BPI evaluates the severity of pain and its impact on functioning. The scores range from 0 to 10 with 0 no pain and 10 the worst possible pain.	X	X	X	X	X
Physical function	PROMIS physical function short form (PROMIS-PF-SF) [21–23] The PROMIS-PF-SF questions a set of person-centered measures that assesses and monitors physical health. The scores range from 1 to 5 with 5 the worst physical function.	X	X	X	X	X
Lymphedema	Lymphedema Symptom Intensity and Distress Survey—Truncal and Head and Neck (LSIDS-H&N) [24] The LSIDS-H&N rates the intensity and distress of 31 symptoms related to HNC (treatment). The scores range from 1 to 5 with 1 slight and 5 severe.	X	X	X	X	X
Fatigue	Functional Assessment of Chronic Illness Therapy—fatigue scale (FACIT-F) [25] The FACIT-F assesses an individual’s level of fatigue and its impact on daily activities and functions. The FACIT-F is scored on a 5-point Likert scale, where some items are reverse scored.	X	X	X	X	X
**Mental function**						
Depression, Anxiety and Stress	Depression, Anxiety and Stress Scale-21 (DASS-21) [26] The DASS-21 is a self-reported questionnaire that measures the three related states of depression, anxiety and stress. The DASS-21 is scored on a 4-point Likert scale, where scores for depression, anxiety, and stress are calculated by summing the scores for the relevant items.	X	X	X	X	X
Self-efficacy	General Self-Efficacy Scale (GSES) [27] The GSES measures how a person generally copes with stressors/difficult situations in life. The scores range from 1 to 4, with a higher score representing a better self-efficacy.	X	X	X	X	X
Stage of readiness to change	Patient-Centered Assessment and Counseling for Exercise (PACE) [28] The PACE determines your current physical activity level. The score ranges from 1 to 11 with 11 representing the most physical active level. The PACE intends to identify the stages of change for physical activity behavior.	X				
**Activity level**						
Physical activity level	International Physical Activity Questionnaire (IPAQ) [29] The IPAQ is a self-reported measure of physical activity. MET minutes a week are calculated to obtain 3 physical activity categories (high, moderate and low) with predefined criteria	X	X	X	X	X
Upper limb function	Short version of the Disability of Arm, Shoulder and Hand (QuickDASH) [30] The QuickDASH is a short self-reported questionnaire on upper limb function. The scores range from 1 to 5 with 5 impossible to perform the activity (and the 2 items “pain” and “pins and needles” with 5 extremely present).	X	X	X	X	X
**Participation**						
Return to work	Self-set questions about return to work, the current work situation, and information about job content	X	X	X	X	X
Social participation	Impact on Participation and Autonomy questionnaire (IPA) [31] The IPA contains questions about activities of daily life, with respect to autonomy and participation. The IPA is scored on a 5-point Likert scale with 0 very good and 5 bad regarding the possibility to perform some specific activities.	X	X	X	X	X
**Exercise**						
Safety	Physical Activity Readiness Questionnaire (PAR-Q) [32] The PAR-Q is designed to help uncover any potential health risks associated with exercise. It is a self-screening tool to determine the safety or possible risks of exercising based on health history, symptoms and risk factors. Yes or no can be answered.	X				
Motivation towards exercise	Behavioral Regulation in Exercise Questionnaire 2 (BREQ-2) [33] The BREQ-2 evaluates behavioral regulation in exercise, in particular motivation to exercise. The BREQ-2 is scored on a 5 point Likert scale (0 = not true for me, 4 = very true for me)	X		X		X
Satisfaction with the supervised intervention at the hospital (IG^a^)	Self-composed set of questions on a 5-point scale about the feasibility (practical, mental, physical), the intensity, the motivation during and the results of the exercise program in the hospital, and the competence of the physiotherapist.			X		
Satisfaction with the intervention at home (IG^a^)	Self-composed set of questions on a 5-point scale about the feasibility (practical, mental, physical), the intensity, the motivation, and the results of the exercise program at home and the added value of the teleconsultations with the physiotherapist.				X	

*T*_0_ = baseline, *T*_1_ = 6 weeks, *T*_2_ = 12 weeks, *T*_3_ = 6 months, *T*_4_ = 12 months

^a^IG = intervention group

**Table 3:** Study outcome measures by assessment time-point: clinical assessments

Domain	Clinical assessment	*T*_0_	*T*_1_	*T*_2_	*T*_3_	*T*_4_
**Physical function**						
Body composition	Bio-impedance Spectroscopy with the InBody device [34, 35] measuring the body composition, with as most important outcome parameters: body mass index (BMI), extracellular water (ECW) ratio, fat free mass, phase angle, and the skeletal muscle index (SMI) to determine sarcopenia.	X	X	X	X	X
External lymphedema	A tissue dielectric constant measurement to determine the %water content at different local points, with the device MoistureMeterD Compact of Delfin [36, 37]. 6 points bilateral in the head and neck area (and 1 point submental) are measured according to the protocol “Lymphscanner/MoistureMeter head and neck area” of C.R. [38] of the Netherlands Cancer Institute (2021).	X	X	X	X	X
External lymphedema	Neck circumference tape measurements at the 3 points in the neck area of the previous protocol [39].	X	X	X	X	X
Fibrosis	Common Toxicity Criteria (CTC) Version 3.0 [40] The CTC grades the severity of possible fibrosis in the acute phase.	X	X	X		
Fibrosis	EORTC/RTOG 5-point scale [41] The EORTC/RTOG grades the severity of possible fibrosis in the more chronic phase.				X	X
Upper limb strength	Jamar Handheld dynamometer [42] This handheld dynamometer measures the handgrip strength.	X	X	X	X	X
Shoulder range of motion	Inclinometer Dr. Rippstein: anteflexion, abduction [43] The active range of motion in the shoulder, anteflexion and abduction, are measured with the inclinometer.	X	X	X	X	X
Physical fitness	6-min walking distance [44] The 6-min walk test assesses aerobic capacity and endurance, giving information about the functional capacity.	X	X	X	X	X
Lower limb strength	Leg press 1 repetition maximum (1RM) The 1RM of the quadriceps is determined during the leg press test, which assesses the maximum muscular strength of the quadriceps.	X	X	X	X	X
**Activity level**						
Physical activity	One ActiGraph wGTX3X-BT accelerometer on the right waist (7 consecutive days) will be worn during waking hours [45], during 7 consecutive days. During sleep, the accelerometer will be worn on the right wrist [46, 47]. Outcome parameters are activity kcals, steps, METs, sedentary time, light vigorous time, moderate vigorous time, vigorous time, and very vigorous time.	X	X	X	X	X

*T*_0_ = baseline, *T*_1_ = 6 weeks, *T*_2_ = 12 weeks, *T*_3_ = 6 months, *T*_4_ = 12 months

#### Feasibility study

The feasibility study exists of a quantitative and qualitative part. First, the quantitative feasibility outcome measures are collected in all participants of the clinical trial, at baseline (T0), 12 weeks (T2), and/or 6 months (T3) follow-up. The outcomes of interest are displayed in Table 4. Second, the qualitative part of the feasibility study consists of organizing focus groups. For the focus group, we anticipate to engage 15 participants out of the total group of 150 participants at each time point (baseline, 12 weeks, 6 months). Review of the literature indicates that data regarding feasibility is typically achieved within 2 to 3 cycles of focus groups [48]. Additionally, data saturation can usually be reached with samples as small as 5 to 7 participants per group. Therefore, we aim to recruit 15 consumers for 2 to 3 focus groups. The aim of these focus groups is to capture qualitative data regarding barriers and facilitators for participating in the CSEP that provided explanation and additional information to the quantitative questionnaire. The focus group leader will ask a series of open-ended questions based predefined subjects. Another researcher will observe the focus groups. All focus group sessions will be video recorded and transcribed.

**Table 4:** Feasibility outcomes: quantitative part

Quantitative outcomes	Explanation	*T*_0_	*T*_1_	*T*_2_	*T*_3_	*T*_4_
Acceptance of the invitation to participate	Eligible patients vs participants	x				
Compliance and adherence to the intervention schedule	For example, adherence to CSEP program, intervention attendance and engagement in scheduled sessions, prescribed frequency, intensity, time, and type of exercise (collected using a diary)			x	x	
Sample characteristics	Sample characteristics based on education level, age, self-efficacy, type of cancer to identify subgroups of head and neck patients who participate, adhere best to the program and would benefit the most of it	x		x	x	
Retention of participants	It is estimated by 6-month follow-up rates				x	

*T*_0_ = baseline, *T*_1_ = 6 weeks, *T*_2_ = 12 weeks, *T*_3_ = 6 months, *T*_4_ = 12 months

### Sample size

The study was powered to have at least 80% power to detect based on a two-sided test with alpha equal to 0.05 a difference of 10 points in general health-related quality of life measured with the EORTC QLQ-C30 (primary outcome) between both groups at the primary endpoint, i.e., 6 months post-diagnosis. A drop-out of 5%, 10%, and 20% is anticipated at 6 weeks, 12 weeks, and 6 months, respectively. Based on reference values for the EORTC QLQ-C30, a standard deviation of 22.7 was assumed [49]. Further, moderate correlations equal to 0.5 were assumed for the associations with the baseline measurement. Under these assumptions, 75 subjects per group are necessary. The study was also powered on one key-secondary outcome, i.e., the physical functioning subscale of the EORTC QLQ-C30. Since the assumed standard deviation for this subscale was slightly lower (based on the EORTC QLQ-C30 reference values 2008), this has no impact on the required sample size. Otherwise said, with 75 subjects per group, the power is higher than 80% for this secondary outcome, more specific 87.7%.

For the feasibility study, no sample size calculation was performed.

### Data analysis

#### Effectiveness study

For the general health-related quality of life and the key-secondary outcome (physical functioning subscale), the mean value at 6 months will be compared between both groups after correction for the baseline value. To handle the presence of missing data, this ANCOVA approach will be implemented using a constrained longitudinal data analysis (cLDA) using all collected measurements over time (baseline, 6 weeks, 12 weeks, 6 months, 12 months). Both comparisons will be based on a two-sided test with alpha equal to 0.05.

#### Feasibility study

All video recorded interviews will be transcribed verbatim. Transcripts (audio and typed feedback) will be uploaded into NVivo 12 coding software. Framework matrix analysis will categorize themes, which will yield specific, recurring information regarding patient feedback [48].

### Data security and management

Data will be prospectively collected from the participating patients by the co-investigators and stored in the Research Electronic Data Capture system (REDCap) [16, 17]. Only the patient number will be recorded in the database. The investigator will maintain a personal patient identification list (patient numbers with corresponding patient names) to enable records to be identified. Clinical patient data will include coded patient-related information, including (but not limited to) age, gender, pathological diagnoses, and clinical TNM stage. The electronic patient file system of the University Hospitals of Leuven will serve as the source for the clinical information, and electronic CRFs will be used for collection of these coded data. A dedicated, trained person will add all research information from this project to the REDCap database, which was specifically designed for this research. All study data will be kept by the principal investigator and the co-investigators. All data uploaded to the cloud system will be coded data; the key of the data will be stored separately from the data. Only coded information will be extracted and used for the downstream research analysis. During the project, research data will be preserved in the shared network drives. After the project, the research data will be preserved in archive drives and external hard disks. All data will be stored for a period of 25 years after the end of the project, according to the requirements of the Clinical Trial Centre of UZ Leuven. All data will be stored on the central servers of UZ Leuven. The final responsibility will be on the principal investigator, SN.

### Trial monitoring

Regarding quality assurance, assessments and interventions will be performed by an experienced physical therapist, KVA, with a master’s degree in rehabilitation sciences. The physical therapist and PI have a GCP certificate. Besides, the PI and UZ Leuven will permit trial-related monitoring, audits, EC review, and regulatory inspections (where appropriate) by providing direct access to source data and other documents.

## Discussion

The clinical trial is ongoing. The recruitment started in January 2022. There are no practical or operational issues involved in performing the study.

### Trial status

This is version 2.2 of the protocol, written on January 20, 2022. The recruitment started in January 26 2022, directly after approval of the Ethics Committee. The approximate date when recruitment will be completed is estimated at the end of 2024.

## Data Availability

The study will be conducted in compliance with the principles of the Declaration of Helsinki (2008) and the principles of Good Clinical Practice and in accordance with all applicable regulatory requirements. The investigator and the participating site shall treat all information and data relating to the study disclosed to the participating site or investigator in this study as confidential and shall not disclose such information to any third parties or use the information for any purpose other than the performance of the study. The collection, processing, and disclosure of personal data, such as patient health and medical information, are subject to compliance with applicable personal data protection and the processing of personal data (European General Data Protection Regulation (GDPR) and the Belgian Law of July 30, 2018, on the protection of natural persons with regard to the processing of personal data). The investigator and the participating site will protect the data from disclosure outside the research according to the terms of the research protocol. Any data required to support the protocol can be supplied on request. The datasets analyzed during the current study and statistical code can be made available by the corresponding author on reasonable request, as the full protocol.

## Abbreviations

EffEx-HN: Effect of exercise in head and neck cancer patients
RCT: Randomized controlled trial
CSEP: Comprehensive supervised exercise program
HNC: Head and neck cancer
CRT: Chemoradiotherapy
QOL: Quality of life
ICF: International Classification of Functioning, Disability and Health
IG: Intervention group
PI: Principal investigator
REDCap: Research Electronic Data Capture system
BPI: Brief Pain Inventory
PROMIS-PF-SF: PROMIS physical function short form
LSIDS-H&N: Lymphedema Symptom Intensity and Distress Survey-Truncal and Head and Neck
FACIT-F: Functional Assessment of Chronic Illness Therapy—fatigue scale
DASS-21: Depression, Anxiety and Stress Scale-21
GSES: General Self-Efficacy Scale
PACE: Patient-Centered Assessment and Counseling for Exercise
IPAQ: International Physical Activity Questionnaire
QuickDASH: Short version of the Disability of Arm, Shoulder and Hand
IPA: Impact on Participation and Autonomy
PAR-Q: Physical Activity Readiness Questionnaire
BREQ-2: Behavioral Regulation in Exercise Questionnaire 2
BMI: Body mass index
ECW: Extracellular water
SMI: Skeletal muscle index
CTC: Common Toxicity Criteria
1RM: One repetition maximum
cLDA: Constrained longitudinal data analysis
ICF: Informed consent form
